# A model for the development of binocular congruence in primary visual cortex

**DOI:** 10.1038/s41598-022-16739-6

**Published:** 2022-07-25

**Authors:** Manula A. Somaratna, Alan W. Freeman

**Affiliations:** Save Sight Institute, Faculty of Medicine and Health, The University of Sydney, Sydney, NSW 2000 Australia

**Keywords:** Network models, Striate cortex

## Abstract

Neurons in primary visual cortex are selective for stimulus orientation, and a neuron’s preferred orientation changes little when the stimulus is switched from one eye to the other. It has recently been shown that monocular orientation preferences are uncorrelated before eye opening; how, then, do they become aligned during visual experience? We aimed to provide a model for this acquired congruence. Our model, which simulates the cat’s visual system, comprises multiple on-centre and off-centre channels from both eyes converging onto neurons in primary visual cortex; development proceeds in two phases via Hebbian plasticity in the geniculocortical synapse. First, cortical drive comes from waves of activity drifting across each retina. The result is orientation tuning that differs between the two eyes. The second phase begins with eye opening: at each visual field location, on-centre cortical inputs from one eye can cancel off-centre inputs from the other eye. Synaptic plasticity reduces the destructive interference by up-regulating inputs from one eye at the expense of its fellow, resulting in binocular congruence of orientation tuning. We also show that orthogonal orientation preferences at the end of the first phase result in ocular dominance, suggesting that ocular dominance is a by-product of binocular congruence.

## Introduction

There is extensive overlap between the monocular visual fields in mammals with frontally placed eyes^1^ and, as a result, many neurons in primary visual cortex receive inputs from both eyes^2,3^. It is well known that receptive field properties of these neurons change little with the eye through which they are stimulated^4,5^. Less is known, however, about the development of binocular cooperation before and after the start of visual experience. Neurons in cat primary visual cortex become orientation selective before the eyes open^6,7^, a property that is probably the result of activity waves passing over each retina^8,9^. Given that wave activity in one eye is largely independent of that in the fellow eye^10^, it might be expected that a neuron’s preferred orientations when driven monocularly will be uncorrelated. Crair et al.^6^ showed, however, that maps of preferred orientation match well between the eyes soon after eye opening. How does binocular congruence of orientation preference develop when the monocular stimuli before eye opening are largely independent?

This question has been partially answered in a recent study^11^ of another carnivore, the ferret. This species is ideally suited to a study of development because there is a relatively long period (about 30 days) between birth and eye opening. Chang et al. mapped orientation preference in primary visual cortex at and after the normal time of opening. The monocular maps were barely correlated before eye opening and highly correlated thereafter. They then tracked orientation preference in individual neurons and showed that the monocular preferred orientations approached one another as a result of visual experience. Similar results have been found in mouse primary visual cortex^12,13^. None of these studies, however, investigated the mechanism by which a neuron’s preferred orientation when driven through one eye can change by up to 90° with visual experience. This is one of the issues that we address in this paper.

Another puzzle is the development of preferred stimulus depth. Each cortical neuron responds most strongly at a specific depth nearer than, at, or beyond the fixation plane. A neuron’s preferred depth is determined by spatial differences in the two monocularly-driven receptive fields^14^. How does this spread of preferred depths develop? A possible answer comes from Sherman^15^, who showed that cats are markedly strabismic when their eyes first open. Perhaps, then, the misalignment of the two eyes after eye opening can train the connections of a cortical neuron in favour of a near or far stimulus. This is the second issue that we investigate here.

We modified an existing computational model of the visual system^16,17^ to address these issues. The model aims to be consistent with known anatomy and physiology and is therefore restricted to a system—the cat’s X-cell pathway under photopic conditions—for which the parameters can be calculated from empirical measurements. Geniculocortical synapses in the model change their strength through Hebbian learning during retinal wave activity before eye opening and through visual experience thereafter. Our aim was to provide a physiologically plausible mechanism for the development of, first, binocular congruence in monocular orientation preferences and, second, a range of preferred depths. A preliminary version of this work has previously appeared as a conference presentation^18^.

## Methods

### Model structure

**Figure 1:**
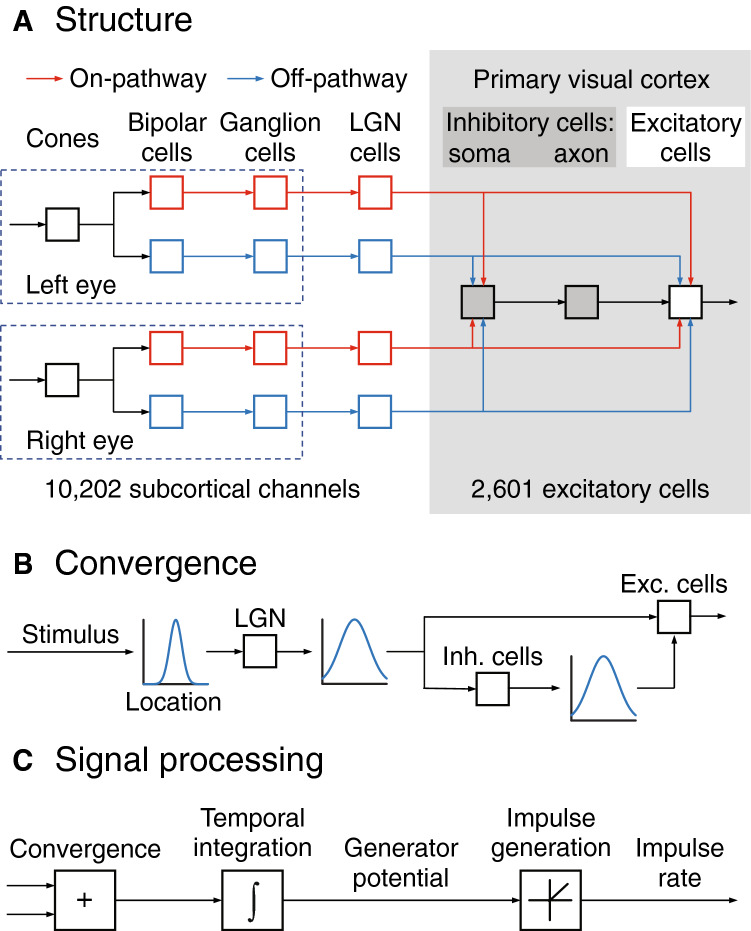
Model elements. (**A**) The subcortical portion of the model comprised multiple channels, with on- and off-centre cells and both eyes represented. All channels converge onto all cortical cells, both inhibitory and excitatory. Inhibitory cells, in turn, converge onto excitatory cells. (**B**) Convergence functions represent the attenuation of a presynaptic signal as a function of its visual field distance from its postsynaptic target. A single function is used for all subcortical sources of convergence and a second, wider, function governs convergence onto excitatory cells from both subcortical and inhibitory sources. (**C**) The model is mathematically defined by a series of differential equations which were numerically integrated to calculate time courses. The flow diagram shows the sequence of signal processing in a typical neuron: convergence of presynaptic signals, integration, and rectification of the generator potential to obtain impulse rate.

The model’s structure is illustrated in Fig. 1A. Visual signals pass serially through four types of subcortical neuron: cones, bipolar cells and ganglion cells in the retina, and lateral geniculate nucleus (LGN) cells in the thalamus. There are multiple channels, also of four types. Each channel passes through either on- or off-centre neurons and originates in either the left or right eye. All channels converge onto each cortical neuron, which is either excitatory or inhibitory. Inhibitory cells are unique in that they have two compartments. The first compartment comprises dendrites and soma, has fast dynamics, and represents the fast-spiking inhibitory cell^19^. The second compartment represents the axon, has slow dynamics, and produces the delayed inhibition seen in neurons presented with flashed stimuli^20^. All inhibitory cells then converge onto each excitatory neuron.

### Neuronal location

**Figure 2:**
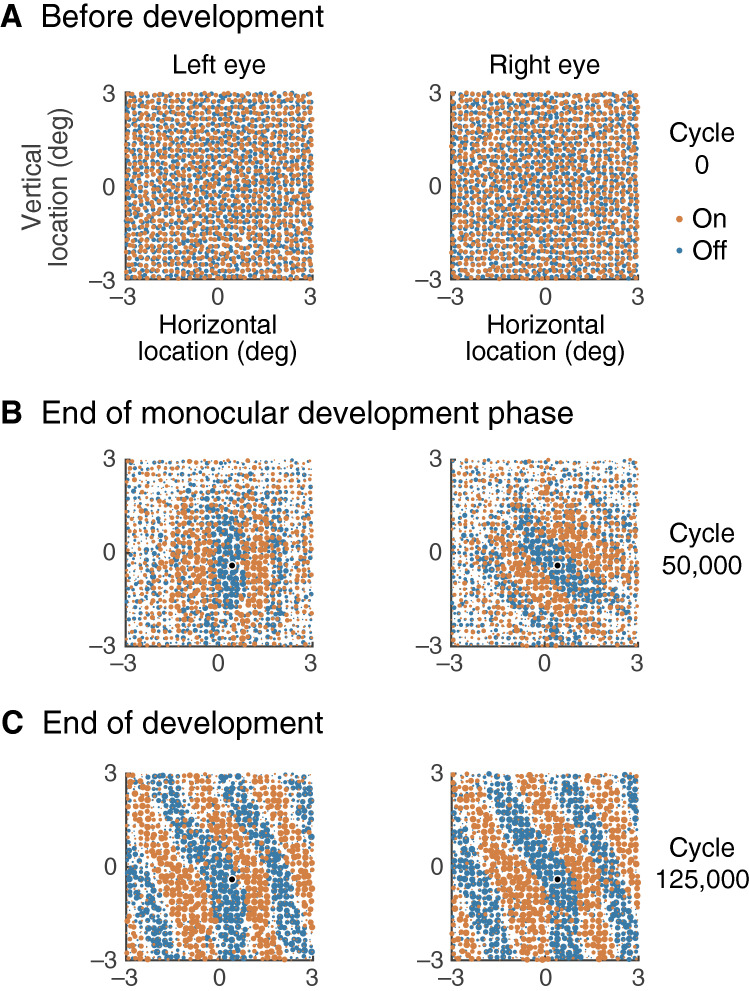
Development process. (**A**) Each dot gives the visual field location of a single subcortical channel, with red and blue for on- and off-centre channels respectively. The spatial arrays are uncorrelated between eyes. (**B**) The plots in (**B**) and (**C**) show geniculocortical synaptic weights for the (excitatory) cortical neuron located at the black dot; red and blue dot diameter indicates the synapse’s modulation factor. Cortical inputs from neighbouring channels of opposite sign tend to cancel each other before development, resulting in very weak cortical signals. Hebbian development in the geniculocortical synapses strengthened cortical responses by segregating on- and off-centre channels. Simulation in the first development phase is monocular, however, so the orientation of segregation is independent between the eyes. (**C**) Stimulation in the second phase of development is binocular, so synaptic modulations adjust to minimise cancellation of one eye’s signals by those from the other eye. Interocular differences in segregation therefore diminish.

All neurons in a subcortical channel are assumed to have the same visual field location. These locations are shown in Fig. 2A, where red and blue dots represent on- and off-centre channels, respectively. The statistics of these locations were taken from a study of ganglion cell arrays in the cat retina^21^; the calculation of model parameters is described below. Off-channels were placed at the nodes of a square grid centred in the visual field and their horizontal and vertical locations were perturbed with a Gaussian deviate. On-channels were placed on a second square grid which was displaced so that the four innermost nodes were equally distant from the middle of the visual field, and the locations were again perturbed. The seed of the random number generator used to calculate one eye’s channel array differed from that for the fellow eye, yielding independent arrays. A square grid was also used for cortical neurons. The grid was centred in the visual field, was unperturbed, and each node was occupied by one excitatory and one inhibitory neuron.

### Convergence

There is convergence between one processing stage and the next at three places in the model, as shown in Fig. 1B. All sources of subcortical convergence—both optical and neuronal—are collapsed into a single convergence function. The remaining sites of convergence are from LGN axons onto cortical dendritic trees, and from inhibitory to excitatory cells. All three convergence functions are shown to scale, and have the same form, as follows. The generator potential, $p$, is the difference between membrane and resting potential in non-spiking cells (cones and bipolar cells) and is the difference between membrane potential and action potential threshold in the remaining cells. Let ${p}_{j}$ be the generator potential in neurons converging on a postsynaptic cell with generator potential ${q}_{i}$. Linear summation is assumed: ${q}_{i}=\sum_{j}{w}_{ij}{p}_{j}$. The synaptic weight ${w}_{ij}$ comprises three factors, ${w}_{ij}={m}_{ij}{a}_{ij}/n$. The first factor, ${m}_{ij}$, is the modulation due to developmental learning. It varies above and below unity in the geniculocortical synapse, as described below, is 1 or more for the inhibitory synapses, and is fixed at 1 for the stimulus-to-subcortical convergence. The attenuation, ${a}_{ij}$, is due to the visual field displacement between the pre- and post-synaptic cells. Each neuron has a receptive field centred on a location ${\varvec{x}}=(x,y)$ in the visual field. The attenuation between the neurons ${p}_{j}$, with locations ${{\varvec{x}}}_{{\varvec{j}}}$, and ${q}_{i}$, with locations ${{\varvec{x}}}_{{\varvec{i}}}$, is assumed to be Gaussian:

1 $${a}_{ij}=\mathrm{exp}\left(-{\left|{{\varvec{x}}}_{{\varvec{i}}}-{{\varvec{x}}}_{{\varvec{j}}}\right|}^{2}/{r}^{2}\right)$$

where $r$ is the radius of the convergence function. The normalising factor, $n$, is determined by assuming that the resting generator potential of a neuron is equal to that of its presynaptic input: ${q}_{\text{rest}}={\sum }_{j}{w}_{ij}{p}_{\text{rest}}= {p}_{\text{rest}}$. Thus

$$\sum_{j}{w}_{ij}=\sum_{j}{m}_{ij}{a}_{ij}/n=1 \; and \; n=\sum_{j}{m}_{ij}{a}_{ij}$$

In summary, then, the weight of the synapse between neurons $i$ and $j$ is

2 $${w}_{ij}={m}_{ij}{a}_{ij}/{\sum }_{j}{m}_{ij}{a}_{ij}$$

### Model equations

Figure 1C shows signal processing in a typical model neuron. Presynaptic signals are collected through the dendritic tree, weighted, and summed. The sum is integrated by the cable-like properties of dendrites and soma before being rectified (for a spiking cell) into an action potential rate at the axon’s initial segment. The differential equation used to simulate generator potential, ${q}_{i}(t)$, in the typical neuron is first order:

$$\tau \frac{d{q}_{i}(t)}{dt}={\sum }_{j}{w}_{ij}{p}_{j}(t)-{q}_{i}(t)$$

where $\tau$ is the integration time constant, $t$ is time, and ${p}_{j}$ is a presynaptic potential. This equation can be understood by viewing the right side as the driving force on generator potential: the more the input exceeds the generator potential, the greater is the rate of change of that potential. Action potential rate is proportional to the rectified generator potential:

3 $$\text{action potential rate = }{k}_{\text{rect}}h\left(p\right), h\left(p\right)=\left\{\begin{array}{c}0, p<0\\ p, p\ge 0\end{array}\right.$$

where ${k}_{\text{rect}}$ is the proportionality factor between potential and rate.

We can now state the equations used to calculate model responses. The equations give the generator potential of neuronal arrays, and it is therefore convenient and efficient to state the equations in terms of vectors and matrices. Thus, a vector of potentials—${p}_{1},{p}_{2},\cdots ,{p}_{k}$—is $${\varvec{p}}={\left[\begin{array}{cccc}{p}_{1}& {p}_{2}& \cdots & {p}_{k}\end{array}\right]}^{T}$$ and a matrix of weights is

$${\varvec{w}}=\left[\begin{array}{ccc}{w}_{11}& \cdots & {w}_{1l}\\ \vdots & \ddots & \vdots \\ {w}_{k1}& \cdots & {w}_{kl}\end{array}\right].$$

where vectors and matrices are shown in bold.

The subcortical driving function, $d$, is the cross-correlation of the subcortical convergence function and the stimulus, $s$. The former is assumed to be Gaussian, with unity integral. Thus:

4 $$d\left({\varvec{x}},t\right)=\frac{1}{\pi {r}_{\text{sub}}^{2}}\mathrm{exp}(-{\left|{\varvec{x}}\right|}^{2}/{r}_{\text{sub}}^{2})\star s({\varvec{x}},t)$$

Evaluating the driving function at the channel locations ${{\varvec{x}}}_{{\varvec{i}}}$ gives the column vector ${\varvec{d}}\left({\varvec{x}},t\right)=[d\left({{\varvec{x}}}_{{\varvec{i}}},t\right)]$, and the equation for the cones is then

5 $$\begin{array}{cc}Cones.& \tau \end{array}\frac{d{{\varvec{p}}}_{\text{cone}}}{dt}={-k}_{\text{sens}}{\varvec{d}}-{{\varvec{p}}}_{\text{cone}}$$

where ${k}_{\text{sens}}$ is contrast sensitivity, the leading minus on the right side indicates that the cone hyperpolarises to light, and ${\varvec{x}}\text{ and }t$ are omitted to improve readability. Bipolar cells receive their input from cones and are either on- or off-centre:

6 $$\begin{array}{ll}Bipolar\;cells.& \end{array} {{\varvec{\tau}}}_{{\varvec{n}}}\circ \frac{d{{\varvec{p}}}_{\text{bipolar}}}{dt}={\varvec{n}}\circ {{\varvec{p}}}_{\text{cone}}-{{\varvec{p}}}_{\text{bipolar}}$$

where the symbol $\circ$ represents element-by-element multiplication, and

$${\tau }_{n}\left(i\right)=\left\{\begin{array}{l}\begin{array}{ll}\tau -\delta & \text{bipolar cell }i\text{ is off-centre}\end{array}\\ \begin{array}{cc}\tau +\delta & \text{bipolar cell }i\text{ is on-centre}\end{array}\end{array}\right.$$

$$n\left(i\right)=\left\{\begin{array}{ll}+1& \text{bipolar cell }i\text{ is off-centre}\\ -1& \text{bipolar cell }i\text{ is on-centre}\end{array}\right.$$

The small increment $\delta$ results in off-dominated cortical responses having lower latency than on-dominated responses^22^. Ganglion cells are driven by bipolar cells and have a resting depolarisation, ${p}_{\text{rest}}$, that produces a resting impulse rate:

7 $$\begin{array}{ll}Ganglion\;cells.& \end{array} {{\varvec{\tau}}}_{{\varvec{n}}}\circ \frac{d{{\varvec{p}}}_{\text{gang}}}{dt}={{\varvec{p}}}_{\text{bipolar}}+{p}_{\text{rest}}-{{\varvec{p}}}_{\text{gang}}$$

Relay cells in the lateral geniculate nucleus are driven by the rectified signal from ganglion cells:

8 $$\begin{array}{ll}Geniculate\;cells.& \end{array} {{\varvec{\tau}}}_{{\varvec{n}}}\circ \frac{d{{\varvec{p}}}_{\text{gen}}}{dt}=h({{\varvec{p}}}_{\text{gang}})-{{\varvec{p}}}_{\text{gen}}$$

The assumption here is that ganglion cell impulse rate is proportional to the rectified generator potential, and that postsynaptic potential in the geniculate cell is proportional to ganglion cell impulse rate. All subcortical proportionality constants are absorbed into the contrast sensitivity ${k}_{\text{sens}}$. An inhibitory neuron in cortex receives convergent input from all geniculate cells:

9 $$\begin{array}{ll}Inhibitory\;somas.& \end{array} \tau \frac{d{{\varvec{p}}}_{\text{soma}}}{dt}={k}_{gc}{{\varvec{w}}}_{{\varvec{g}}{\varvec{c}}}h\left({{\varvec{p}}}_{\text{gen}}\right)-{{\varvec{p}}}_{\text{soma}}$$

where ${k}_{\text{gc}}$ is geniculocortical gain and ${{\varvec{w}}}_{{\varvec{g}}{\varvec{c}}}$ is the matrix of connection weights between the geniculate and cortex. The inhibitory axon has a long time constant, ${\tau }_{\text{inh}}$:

10 $$\begin{array}{ll}Inhibitory\;axons.& \end{array} {\tau }_{\text{inh}}\frac{d{{\varvec{p}}}_{\text{inh}}}{dt}=h\left({{\varvec{p}}}_{\text{soma}}\right)-{{\varvec{p}}}_{\text{inh}}$$

Finally, the excitatory neuron has both subcortical and inhibitory input:

11 $$\begin{array}{ll}Excitatory\;cells.& \end{array}\tau \frac{d{{\varvec{p}}}_{\text{exc}}}{dt}={k}_{gc}{{\varvec{w}}}_{{\varvec{g}}{\varvec{c}}}h\left({{\varvec{p}}}_{\text{gen}}\right)-{k}_{ie}{{\varvec{w}}}_{{\varvec{i}}{\varvec{e}}}h\left({{\varvec{p}}}_{\text{inh}}\right)-{{\varvec{p}}}_{\text{exc}}$$

where ${k}_{ie}$ is inhibitory-excitatory gain and ${{\varvec{w}}}_{{\varvec{i}}{\varvec{e}}}$ is the convergence matrix from inhibitory neurons to an excitatory cell. Equations (1)–(11) together define the model.

### Stimuli

#### Drifting grating

The stimulus used for most analyses in this paper was a drifting sinusoidal grating. Model responses were most efficiently and accurately calculated when the cross-correlation in Eq. (4) was solved analytically, and we now provide the solution when the stimulus $s({\varvec{x}},t)$ is a drifting grating. The cross-correlation at location ${{\varvec{x}}}_{i}$ is

12 $$d\left({{\varvec{x}}}_{i},t\right)=\frac{1}{\pi {r}_{\text{sub}}^{2}}{\iint }_{-\infty }^{\infty }\mathrm{exp}(-{\left|{\varvec{x}}\right|}^{2}/{r}_{\text{sub}}^{2})s({{\varvec{x}}}_{i}+{\varvec{x}},t)dxdy$$

Assume that the grating’s direction is $\theta$ and define new spatial variables

13 $${\varvec{u}}=\left[\begin{array}{c}u\\ v\end{array}\right]=\left[\begin{array}{cc}\mathrm{cos}(\theta )& \mathrm{sin}(\theta )\\ -\mathrm{sin}(\theta )& \mathrm{cos}(\theta )\end{array}\right]\left[\begin{array}{c}x\\ y\end{array}\right]$$

Then $u$ is distance in the direction of motion, and the grating is given by

14 $$s\left({\varvec{u}},t\right)=c\cdot \mathrm{cos}(\psi u-\omega t)$$

where $c$, $\psi$ and $\omega$ are grating contrast, spatial frequency, and temporal frequency, respectively. To change the cross-correlation to the new spatial variables, we need the inverse transform

$${\varvec{x}}={\varvec{z}}{\varvec{u}}\text{, where }{\varvec{z}}=\left[\begin{array}{cc}\mathrm{cos}(\theta )& -\mathrm{sin}(\theta )\\ \mathrm{sin}(\theta )& \mathrm{cos}(\theta )\end{array}\right]$$

Then $${\left|{\varvec{x}}\right|}^{2}={{\varvec{x}}}^{T}{\varvec{x}}={{\varvec{u}}}^{T}{{\varvec{z}}}^{T}{\varvec{z}}{\varvec{u}}={{\varvec{u}}}^{T}u={\left|{\varvec{u}}\right|}^{2}$$, and

$$dxdy=\left|\begin{array}{cc}\partial x/\partial u& \partial x/\partial v\\ \partial y/\partial u& \partial y/\partial v\end{array}\right|dudv=dudv$$

For location ${u}_{i}$, the driving function then becomes

$$d\left({u}_{i},t\right)=\frac{c}{\pi {r}^{2}}{\iint }_{-\infty }^{\infty }\mathrm{exp}(-({u}^{2}+{v}^{2})/{r}_{\text{sub}}^{2}){\text{cos}}\left(\psi \left({u}_{i}+u\right)-\omega t\right)dudv$$

Expanding the cosine term and removing the sine term (because it is odd-symmetric):

15 $$ \begin{aligned} d\left( {u_{i} ,t} \right) & = \frac{c}{{\pi r^{2} }}\int\limits_{ - \infty }^{\infty } {\cos \left( {\psi u} \right){\text{exp}}( - u^{2} /r_{{{\text{sub}}}}^{2} )du} \int\limits_{ - \infty }^{\infty } {{\text{exp}}( - v^{2} /r_{{{\text{sub}}}}^{2} )dv} \cos \left( {\psi u_{i} - \omega t} \right) \\ & = c \cdot {\text{exp}}( - r_{{{\text{sub}}}}^{2} \psi^{2} /4)\cos \left( {\psi u_{i} - \omega t} \right) \\ \end{aligned} $$

#### Sparse noise

The stimulus we used to map receptive fields was a briefly presented square of light or dark:

16 $$ s\left( {{\varvec{x}},t} \right) = \left\{ {\begin{array}{*{20}l}    {c,} \hfill & {x\;{\text{within}}\;{\text{square}}\;{\text{and}}\;t\;{\text{within}}\;{\text{presentation}}\;{\text{time}}} \hfill  \\    {0,} \hfill & {{\text{otherwise}}} \hfill  \\   \end{array} } \right. $$

Squares had a width of 1° and were presented on a square grid of visual field locations with spacing 0.25°. Stimulus duration was 0.05 s and contrast $c=1$ for light squares and $c=-1$ for dark.

### Development

Development in the model occurred through Hebbian plasticity in the synapses connecting geniculate neurons to the cortex. Each synapse was assigned a modulation factor, $m$, of 1 before development started. Development then proceeded in cycles: on each cycle 0.2 was added to the modulation factor for every axonal terminal of a randomly selected subcortical channel. The model was then stimulated with gratings drifting in 16 directions evenly distributed across the full range. If the maximum response of a cortical neuron was larger than on the previous cycle, the modulation factor of its synapse with the selected channel was retained. Otherwise, the factor was decreased by 0.2 from its value at the end of the previous cycle. Modulation factors were restricted to lie between 0 and 2.

Development proceeded in two phases. In the first, designed to simulate events prior to eye opening, stimulation was restricted to the eye supplying the selected channel; stimulus contrast was set equal to 0 for the other eye. Stimulation was chosen to be monocular because measurements from mouse retina before eye opening show simultaneous binocular activity only about 16% of the time^10^. The second phase followed eye opening and therefore used simultaneous stimulation of both eyes. In this case, however, the right eye’s stimulus was spatially offset to simulate variability in binocular fixation. The offset took 5 values distributed evenly from − 0.5 to 0.5 deg (degrees of visual angle) measured perpendicularly to grating bars. This range was chosen to be wider than that found in the laboratory^23^. All 5 values were used on each development cycle, and the maximum of a cortical response across all stimulus directions and offsets then determined changes in modulation factor.

The model contains 10,202 subcortical channels across its $10^\circ \times 10^\circ$ visual field, and each channel was selected an average of 5 times to allow each of its synapses to reach a modulation factor of 0 or 2. The number of cycles in the first phase was therefore set at 50,000 (by rounding $\mathrm{10,202}\times 5$). The second phase used 75,000 cycles because trial and error showed that this number was required to obtain responses close to their asymptotic values (Fig. 4C). There was one more component of development. Inhibitory synapses change during development^24^, preventing Hebbian plasticity from incrementing response amplitude without limit. We therefore increased modulation factors for the synapses from inhibitory to excitatory cells; the increase was from 1 to ${k}_{ie}$, and linear with cycle number in the first phase.

### Parameter settings

Parameter settings and a glossary of symbols are shown in Table 1. Parameters were set where possible from published values and are the same, with a few exceptions, as in Nguyen and Freeman^16^. The exceptions are now listed and explained.

**Table 1:** Glossary of symbols and model parameters.

Symbol	Function or parameter	Value	Source
${\varvec{a}}$	Convergence attenuation	Variable, dimensionless	Equation (1)
$c$	Stimulus contrast	0.3 contrast-units, unless otherwise stated	
$d$	Driving function	Contrast units	Equation (4)
$h$	Rectification function		Equation (3)
${k}_{gc}$	Geniculocortical gain	7	Carandini et al.^25^
${k}_{ie}$	Inhibitory-excitatory gain	1.66	Anderson et al.^26^
${k}_{\text{rect}}$	Rectification constant	7.2 Hz/mV	Carandini et al.^25^
${k}_{\text{sens}}$	Contrast sensitivity	62 mV/contrast-unit	Frishman et al.^27^, Kaplan et al.^28^
${\varvec{m}}$	Synaptic modulation factor	0 to 2 for geniculate-cortex, 1 to ${k}_{ie}$ for inhibitory-excitatory synapse	Methods
$\omega$	Grating temporal frequency	$\left(2\text{ Hz}=\right) 2\pi \times 2$ radians/s	
${\varvec{p}}$	Generator potential	Variable, unit is mV	
${p}_{\text{rest}}$	Resting generator potential	1.9 mV	Kaplan et al.^28^
$\psi$	Grating spatial frequency	(0.5 cycles/deg =) $2\pi \times 0.5$ radians/deg	
${r}_{\text{cort}}$	Cortical convergence radius	0.95 deg	Jones et al.^29^
${r}_{\text{sub}}$	Subcortical convergence radius	0.4 deg	Saul et al.^30^
$s$	Stimulus	Variable, contrast units	
$t$	Time	Variable, unit is second	
$\tau$	Time constant	0.01 s	Nguyen et al.^16^
${\tau }_{n}$	Subcortical time constant	$$ \left\{ {\begin{array}{*{20}l} {0.0095{\text{ s}}} \hfill & {{\text{off{-}centre}}} \hfill \\ {0.0105{\text{ s}}} \hfill & {{\text{on{-}centre}}} \hfill \\ \end{array} } \right. $$	Komban et al.^22^
${\tau }_{\text{inh}}$	Inhibitory cell time constant	0.1 s	DeAngelis et al.^20^
$\theta$	Grating direction	Variable, unit is radian	
${\varvec{u}}$	Visual field location, aligned with grating direction	Variable, unit is deg	Equation (13)
${\varvec{w}}$	Synaptic weight	Dimensionless	Equation (2)
${\varvec{x}}$	Horizontal and vertical location in visual field	Variable, unit is deg	

The table show the symbols used for model parameters and functions, parameter values where relevant, and the equations or published papers used to calculate parameter values. Bold symbols represent vectors and matrices.

#### Cortical cell spacing

Cortical cells were located on a square grid with an excitatory and an inhibitory neuron at each node. Horizontal and vertical spacing between nodes was 0.2 deg. The only functional requirement on this value is that it be substantially less than the radius of cortical convergence, ${r}_{\text{cort}}$.

#### Time constants

The difference between on-centre and off-centre cell time constants is set equal to 1 ms. This replicates the latency difference between cortical inputs of opposite sign^22^. The time constant for inhibitory axons in the model is 100 ms, which is consistent with the timing of rebound responses to flashed stimuli^20^ and the delay of inhibitory conductance relative to excitatory conductance during cyclic stimulation^31^.

#### Geniculocortical gain

This parameter was estimated from intracellular recordings in cat simple cells stimulated monocularly with optimally oriented drifting gratings^25^ (Fig. 13): averaging the maximum contrast sensitivity across three cells gives an average of 70 mV/contrast-unit. The gain ${k}_{\text{gc}}$ was set so that model excitatory cells closely reproduced this value.

#### Inhibitory-excitatory gain

This gain, ${k}_{ie}$, determines resting hyperpolarisation in cortical excitatory cells. Anderson et al.^26^ measured this quantity in nine simple cells and found a median difference of 9 mV between resting and threshold potential; the gain was therefore set by solving the equation ${k}_{\text{gc}}\times {p}_{\text{rest}}\left(1-{k}_{ie}\right)=-9$.

### Analysis

#### Curve fitting

The curves fitted to the direction tuning data in Figs. 4B and 7A used a sum of von Mises functions:

17 $$r={r}_{0}+{r}_{p}\mathrm{exp}(k(\mathrm{cos}(\theta -{\theta }_{p})-1))+{r}_{s}\mathrm{exp}(k(\mathrm{cos}(\theta -{\theta }_{s})-1))$$

where $r$ is impulse rate, ${r}_{0}$ is resting rate, $p$ and $s$ refer to the preferred and suboptimal motion directions, $k$ is a constant which determines tuning bandwidth, and $\theta$ is motion direction. For the disparity tuning curves in Fig. 6A, the equation is

18 $$r={r}_{0}+{r}_{p}\left|\mathrm{cos}(\psi (u-{u}_{p})/2)\right|$$

where $\psi$ is stimulus spatial frequency, $u$ is visual field location in the grating motion direction, and $p$ refers to preferred disparity.

#### Ocular dominance

The ocular dominance index was calculated from the response to a drifting grating optimised for the dominant eye: ${\text{ODI}}={r}_{R}/({r}_{L}+{r}_{R})$ where ${r}_{L}$ and ${r}_{R}$ are the maximum impulse rates when stimuli were delivered via the left and right eyes, respectively. Monocularity is $2\left|{\text{ODI}}-0.5\right|$.

### Computation

Model responses were computed in two ways: by numerical integration of the differential equations, and in the frequency domain^16^. The results from the two methods were compared to ensure that they matched within roundoff error, reducing the possibility of computational error.

## Results

### The model

We investigated the development of binocular congruence using a computational model. The model’s circuit diagram is summarised in Fig. 1A. There are multiple subcortical channels, each of which passes through either on- or off-centre cells and derives from either eye. Visual signals in each channel pass serially through cones, bipolar cells, a ganglion cell, and a relay cell in the lateral geniculate nucleus. Subcortical activity then converges onto excitatory neurons in layer 4 of primary visual cortex. The same activity also converges onto inhibitory neurons, which each consist of two compartments. The first, which comprises dendrites and soma, simulates the fast-spiking interneuron^19^ and the second compartment, the axon, accounts for the long-lasting inhibitory tail seen in simple cells stimulated with flashed stimuli^20^.

Signals converge at three sites in the model as shown in Fig. 1B. All subcortical convergence is combined into a single synaptic weighting, the subcortical convergence function. The second convergence function represents subcortical input to excitatory and inhibitory cortical neurons. Finally, inhibitory neurons converge onto excitatory neurons in a third function. Signal processing in an individual neuron is shown in Fig. 1C. Synaptic inputs are weighted, summed, and integrated over time as the signal passes from dendrites to the axon initial segment. In spiking neurons, membrane potential is then rectified to produce an impulse rate. Each neuron was represented with a differential equation, and all equations were numerically integrated to provide the model’s response to a stimulus. Equations and parameter values are provided in the Methods.

### Development

Figure 2 maps the spatial locations of subcortical channels within the visual field. Part A of the figure illustrates the retinal ganglion cell array, with the two retinas shown at left and right. On- and off-centre cells are shown in red and blue, respectively. The spatial arrays simulate those described by Wässle et al.^21^ and reproduce the statistical properties of the empirical measurements^16^.

The development process consisted of a series of cycles. On each cycle a channel was randomly chosen from either eye, and its synaptic weights onto all cortical neurons was increased. The model was then stimulated with gratings drifting across the full range of directions and the maximum response of all cortical excitatory neurons calculated. If the response in a neuron increased, the synapse between the channel and neuron retained its increased weight, otherwise the weight was reduced below its original value. Synaptic weight is the product of two factors: an attenuation that depends on the visual field distance between the pre- and post-synaptic neurons, and the modulation resulting from development. The modulation factor is shown in the figure by the diameter of the dot representing each channel.

There were two phases in the development process. The first phase simulated development before eye opening, when cortical stimulation results from retinal activity waves. Here the stimulus on each cycle was delivered to only the eye containing the channel for which synaptic weight was changed. The stimulus was restricted to one eye because it has been shown that retinal waves in the newborn mouse usually occur in the absence of activity in the fellow eye^10^. The second phase simulated development after eye opening, when stimulation is visual. In this case stimuli were delivered to both eyes; the monocular stimuli were the same except for a randomised difference in spatial phase.

Cortical responses were very weak at the start of monocular stimulation because neighbouring on- and off-channel signals tended to cancel each other when summed at a cortical neuron. The Hebbian development process strengthened the cortical response by increasing the weights of one channel at the expense of neighbouring opposite-sign channels. The channel sign that dominated was that with a local density higher than that of opposite-sign channels, as shown in a previous version of the model^16^. The result at the end of monocular development is shown in Fig. 2B. These maps show the modulation factor of the synapse between each geniculate cell and the cortical neuron whose location is shown by the black dot. As shown previously^16^ development segregates on- and off-channels into oriented bands resembling a simple cell receptive field. The orientation, however, differs between the eyes.

The second phase of development simulates the period after eye opening, when stimuli are binocular; its result is shown in Fig. 2C. Early in this phase signals from visual field areas dominated by on-centre channels in one eye cancel those from off-centre areas in the other eye, resulting in relatively weak cortical signals. Hebbian development favours one eye over the other in each area, producing synaptic modulation bands whose orientations are much better aligned between the eyes. We propose that this is the basic mechanism for the development of binocular congruence.

**Figure 3:**
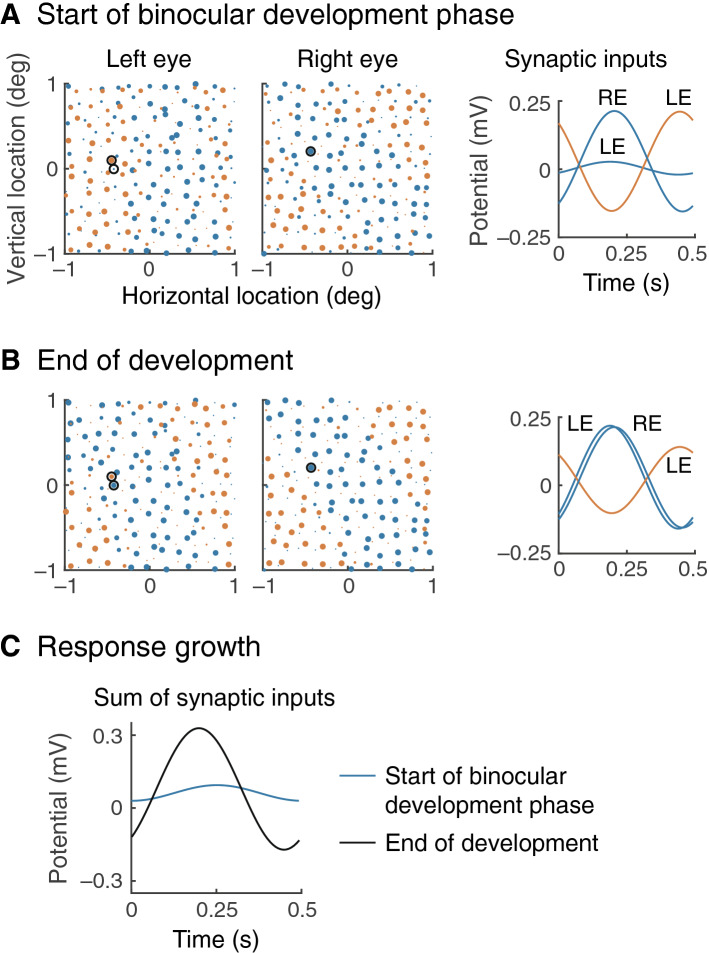
Mechanism of binocular congruence. (**A**) The visual field maps at left are expanded versions of Fig. 2B. Three subcortical inputs are highlighted by black circles. The graph at right shows the time course of these inputs in response to a drifting grating; LE and RE stand for left and right eye, respectively. There is destructive interference between the responses when added at the synapse with the cortical neuron. (**B**) Hebbian plasticity during the binocular phase of development up- and down-regulates the synaptic weights of circled left-eye off- and on-channels, respectively. (**C**) These synaptic changes improve the amplitude of the summed synaptic inputs.

Figure 3 illustrates this mechanism in more detail. The maps in part A are magnified from those in Fig. 2B, and therefore show the strength of the geniculocortical synapses at the start of the binocular development phase. The black circles surround three representative subcortical inputs, and the graph at right shows their responses to a grating drifting in the preferred direction. The grating is presented to both eyes, so the responses are summed at the cortical neuron’s dendrites. Given that the right-eye off-centre response is inverted relative to the left-eye on-centre response, there is mutual cancellation as shown by the weak summed response in part C. At the end of development, shown in part B, Hebbian plasticity has strengthened the left-eye off-centre input and weakened the on-centre input, producing a much stronger summed input at the cortical cell. In the process, the orientation of off-centre segregation in the left eye has swung around to match the right eye, as can be seen from the maps in part B.

### Receptive fields

**Figure 4:**
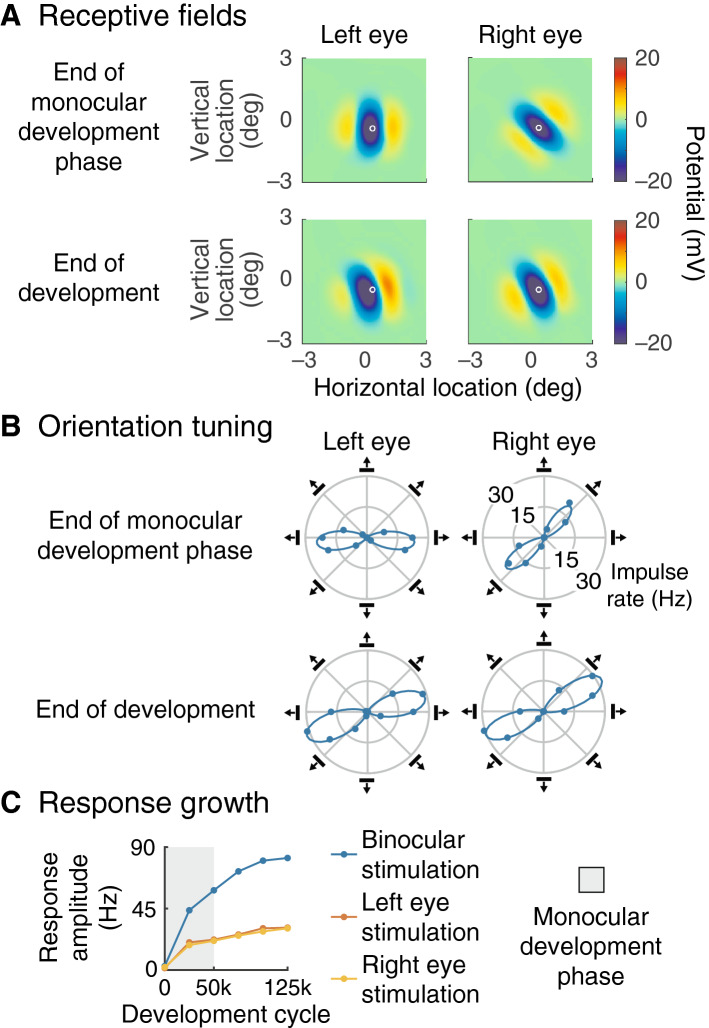
Binocular development in a single neuron. (**A**) This shows receptive fields for the neuron whose synaptic modulations are given in Fig. 2; the white circle shows the neuron’s location. Fields were calculated by presenting a square of light or dark at a range of visual field locations, subtracting responses to dark from those to light, and interpolating across locations. Generator potential was used to represent the response because it reveals decrements as well as increments. The left and right plots are for stimuli delivered to the left and right eye respectively. The orientation of the subfields at the end of the first (upper row) and second (lower row) phases of development reflects the orientation of the synaptic modulation segregation in Fig. 2B,C, respectively. (**B**) To illustrate orientation tuning, gratings with a variety of directions were drifted over the receptive fields of the same neuron as in (**A**). The polar plots give the fundamental Fourier amplitude of impulse rate: amplitude is shown by distance from the origin, and direction by the angle of the plotted point from the horizontal. The icons around the plot show both the orientation of the grating and its motion direction, and the curve fitted to the points is a sum of von Mises functions. There is a clear misalignment of preferred orientation between the eyes at the end of the monocular development phase (upper row). The left and right eye orientation tunings are almost aligned by the end of the second development phase (lower row). (**C**) The horizontal axis gives development cycle number and the vertical axis the response amplitude at the preferred orientation. The two lower curves show responses for monocular stimulation and the upper (blue) curve for simultaneous stimulation of both eyes. The first development phase is shown by the grey shading. Response amplitudes are very weak at the start of development and are close to asymptotic at the end.

The synaptic modulation maps are suggestive of receptive fields. We calculated receptive fields by presenting squares of light and dark at a variety of visual field locations. The time course of the generator potential evoked in response to a dark stimulus was subtracted from that to light, and the maximum over time was used to give the response at each location. Figure 4A shows receptive fields for the cortical neuron whose synaptic modulation maps are shown in Fig. 2. Fields for left and right eye stimulation are shown on the left and right, respectively, and for two stages of development—end of monocular stimulation and end of development—above and below. The receptive fields are smaller than the synaptic modulation maps because inputs from the outer parts of the maps are strongly reduced by distance-based attenuation, but the orientations in the maps are reflected in the fields.

### Orientation tuning

The receptive fields in Fig. 4A are like those of simple cells and therefore predict that post-development neurons in the model will be orientation selective. We tested orientation tuning with drifting gratings delivered across the full range of orientations. Figure 4B shows the results for the neuron illustrated in part A. The graphs are polar so that the angle of the line joining a point to the origin represents grating direction (as shown by the grating icon) and the distance from the origin gives response amplitude (measured as the fundamental Fourier amplitude of impulse rate). The curved line is a fitted sum of two von Mises functions as described in the Methods. At the end of monocular stimulation, the neuron’s preferred orientations for left and right eye stimulation differ by about 45°, as shown by the upper pair of graphs. At the end of development, shown in the lower graphs, the interocular difference in preferred orientations has decreased to about 10°. For this neuron, then, binocular congruence of orientation is much improved.

Figure 4C shows how responses grow during development. The vertical axis gives response amplitude when the cortical neuron illustrated in parts A and B is stimulated with an optimally oriented grating either monocularly or binocularly. There are three things to note in this graph. First, responses are very weak before development. Second, response growth slows towards the end of the monocular development phase (shown by the shaded area). Third, responses grow again in the binocular phase of development and approach asymptotic levels. As expected of Hebbian plasticity, therefore, responses grow throughout the development process.

**Figure 5:**
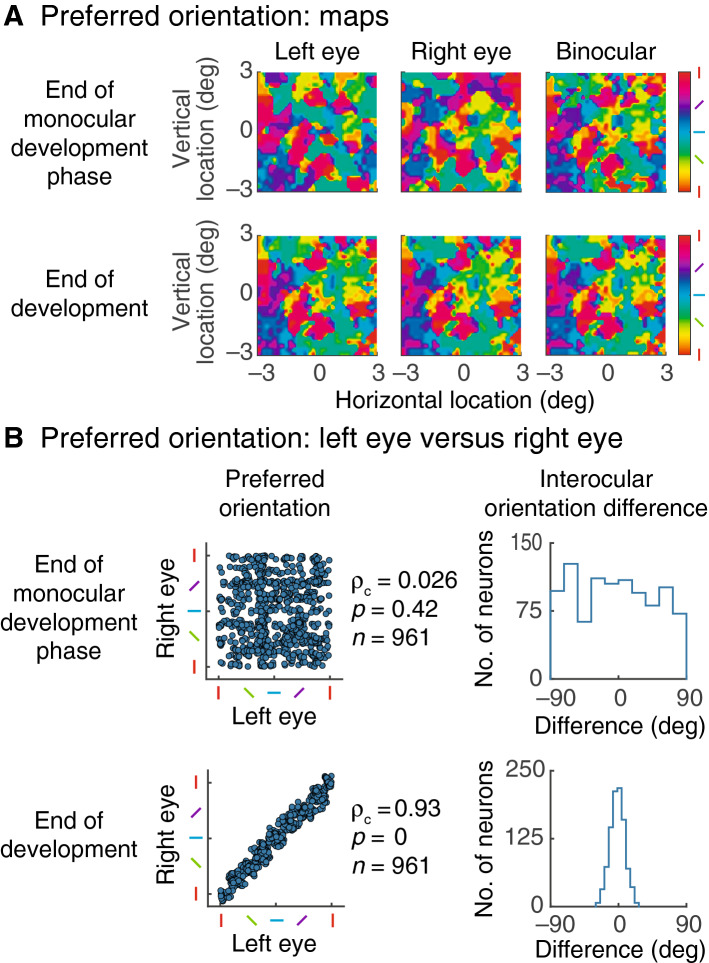
Binocular development across the neuronal population. (**A**) Orientation tuning was measured for all (excitatory) cortical cells and the preferred orientations are colour coded for each visual field location. The colour key is shown at the right. There is no match between orientation preference for left and right eye stimulation at the end of the monocular development phase (upper row). The map resulting from binocular stimulation, shown at right, contains elements of both monocular plots. By the end of the second development phase the left and right eye maps are very similar, as shown in the lower row. As expected, binocular stimulation at the end of development (right plot) gives a map that is like both the monocular plots. (**B**) This shows interocular matching of orientation preference. The left side shows, on the horizontal and vertical axes, preferred orientation for stimuli delivered to the left and right eye respectively; each neuron is represented by a single dot. At the end of the monocular phase of development (upper row) there is no clear interocular relationship. Correspondingly the histogram (at right), which gives the difference between left and right eye preferred orientations, is essentially flat. At the end of the binocular development phase (lower row) left and right eye orientation preferences are now similar (left graph) and the histogram of interocular orientation difference (right graph) deviates little from zero.

The conclusions we have drawn from a single cell extend to the whole population of excitatory neurons in the model. Figure 5A shows maps of preferred orientation across the visual field. Responses were calculated for a $10^\circ \times 10^\circ$ visual field but only $6^\circ \times 6^\circ$ are displayed, to avoid edge effects. For each neuron, orientation preference was taken to be the orientation at which response amplitude was maximum in tuning curves such as those in Fig. 4B. The top left and middle maps were obtained at the end of the monocular phase of development via stimulation of the left and right eyes, respectively. Visual inspection reveals little resemblance between the maps. Indeed, the correlation between them is not significant, as shown in Fig. 5B. The left and middle maps in lower Fig. 5A were calculated at the end of development and appear similar: the two maps in this case are highly correlated (see Fig. 5B for the statistics).

A final comparison between maps is shown at the right of the figure. These two maps came from the end of the first (upper) and second (lower) phases of development but were both obtained with binocular stimulation. Comparison with the previous maps shows that the upper map contains elements of the upper monocular maps and that the lower map is highly correlated with the lower monocular maps (end of monocular phase, binocular versus left eye: ${\rho }_{c}=0.40,p=0,n=952$; end of development, binocular versus left eye: ${\rho }_{c}=0.96,p=0,n=961$; similar statistics for right eye). The statistical tests here and for Fig. 5B were performed using the circular correlation coefficient, ${\rho }_{c}$, because the data are cyclic^32^. These findings match the evidence from Chang et al.^11^ that, first, there are three distinct maps of preferred orientation when the eyes first open and, second, all three maps are correlated after development.

A more direct way of displaying interocular orientation difference is shown in upper Fig. 5B, which illustrates the end of the monocular development phase. The horizontal and vertical axes on the left give the preferred orientation when a neuron is stimulated through the left and right eyes, respectively, with each neuron represented by one dot. Correlation between these quantities is not significant (${\rho }_{c}=0.026,p=0.42,n=961$). The graph on the right shows a histogram of interocular orientation difference which, as expected, is basically flat. For lower Fig. 5B, representing the end of development, the interocular correlation is highly significant (${\rho }_{c}=0.93,p=0,n=961)$ and the graph at right is well approximated by a Gaussian probability density. The standard deviation here is 9.6 deg which approximates an empirical measurement, 6.7 deg, of the interocular difference in preferred orientations^4^.

### Binocular disparity

While the binocular congruence of orientation tuning is an essential contributor to visual sensitivity, there is also an important binocular incongruence. Binocular disparity is the interocular difference in retinal location of a viewed object. Cortical neurons can be selective for this disparity because one monocular receptive field is displaced relative to the other; this selectivity is thought to lay a foundation for depth perception^33^. Consistent with the finding that the species modelled here (cat) is strabismic during early visual experience^15^, we simulated strabismus during development by spatially offsetting gratings presented to one eye relative to those to the fellow eye. As a result, the receptive field of a neuron stimulated through one eye can be displaced relative to that for the other eye, as shown by the example in lower Fig. 4A. The range of offsets used during development, − 0.5 to 0.5 deg, was greater than the range of receptive field disparities found in published work^23^. The full range of orientations and offsets was presented on every development cycle.

**Figure 6:**
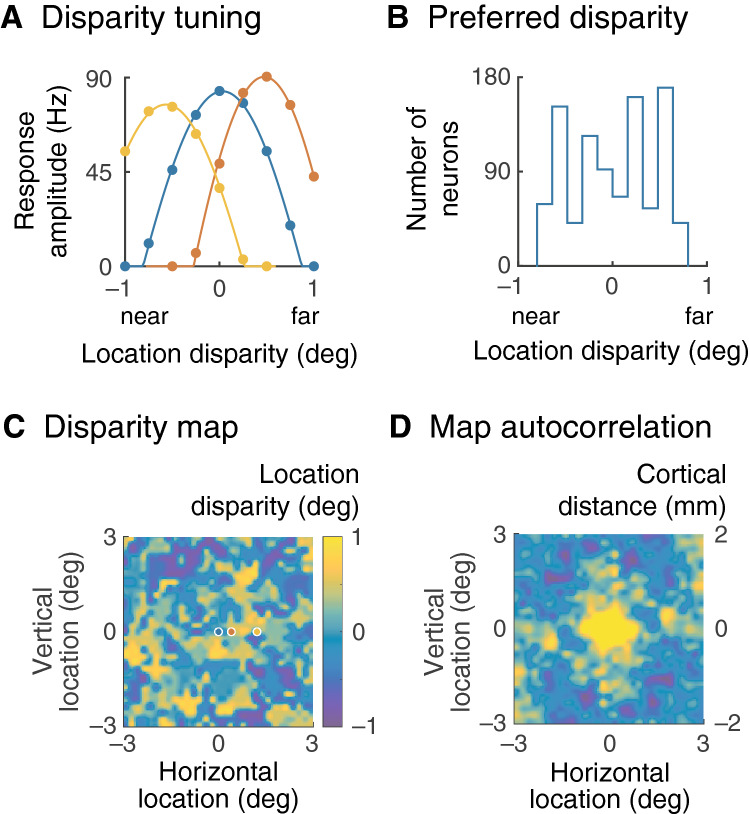
Binocular disparity. The stimuli during the binocular phase of development were spatially offset to simulate instability in binocular fixation. This resulted in neurons that differed in their preferred binocular disparities. (**A**) Disparity tuning is shown for three cells, in differing colours, to exemplify tuning for points nearer and further than the fixation plane, and on the plane. Cell location is shown in (**C**) of the figure. Disparity is measured as visual field distance perpendicular to grating bars. The stimuli were cyclic in time, and the response is therefore also cyclic; only one half-cycle of the response is shown. The stimuli were also cyclic in space, and the horizontal axis spans one period of the stimulus. The fitted curve is a rectified sinusoidal function, as described in the Methods. (**B**) The histogram of preferred disparity shows a spread across almost two degrees of location disparity, consistent with empirical findings. (**C**) Mapping preferred disparity across the visual field shows clusters of cells that prefer either near or far disparities, suggesting a columnar structure. (**D**) Periodicity in preferred disparity was measured with the autocorrelation of the map in (**C**). The conversion from degrees of visual angle, left axis, to cortical distance is shown on the right axis. The light areas around the central peak indicate a mean periodicity of 1.0 mm.

Quantitative examples are shown in Fig. 6, which gives model data after development. Part A of the figure shows disparity tuning for three representative cells. Optimally oriented gratings were drifted over their receptive fields with a range of phase offsets between the two eyes, as shown on the horizontal axis. The vertical axis gives the fundamental Fourier amplitude of impulse rate. The three cells were chosen to show preferred disparities corresponding to optimal stimulation nearer than, at, and further than the fixation point. Figure 6B shows a histogram of preferred disparity for all neurons in the visual field. There is a spread of preferred disparity with a standard deviation of 0.43°, comparable with an empirical measurement, 0.59°^23^.

We next asked whether preferred disparity is distributed periodically across the visual field, as with preferred orientation and ocular dominance. Figure 6C gives a map of preferred disparity, which shows clustering of neurons tuned for either near or far disparities. To test for periodic variation, we autocorrelated the map as shown in part D of the figure. This map was averaged across three simulations for the model, using a different seed for the random number generator on each run. All autocorrelations have a maximum at the origin, which can therefore be ignored. The remaining maxima closest to the origin are an average of 1.6 deg from the origin which, for the eccentricity modelled here (11° temporal), corresponds to 1.0 mm of cortical distance (see the vertical axis on the right). This value is close to the width, 1–1.1 mm, measured for orientation hypercolumns in primary visual cortex^34^ and to the median periodicity of binocular disparity, 0.96 mm, found in macaque area V2^35^.

### Ocular dominance

**Figure 7:**
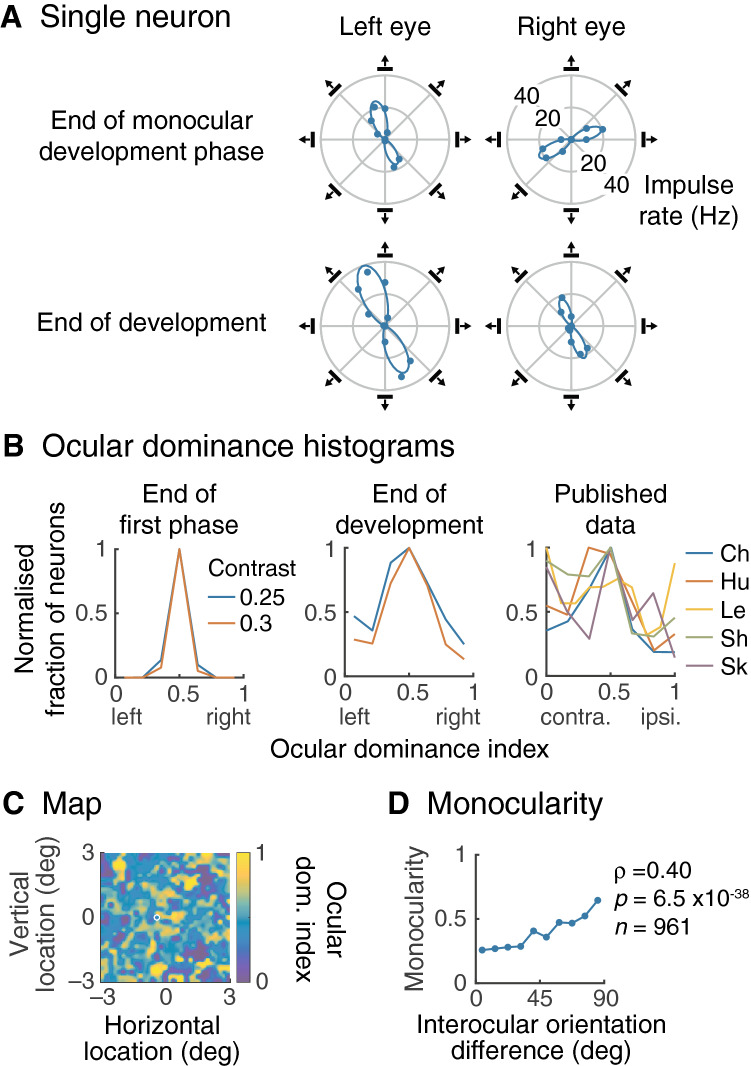
Ocular dominance. (**A**) At the end of the monocular development phase each neuron produces much the same response amplitude whether stimulated via the left or right eye. In the case of the neuron illustrated, the similarity of response amplitude is in contrast with the preferred orientations, which are close to orthogonal (upper row). The neuron’s location is shown by the white circle in (**C**). At the end of development (lower row), the preferred orientations are now aligned for left and right eye stimulation, but response amplitude has improved much more for the left eye. That is, the cell is now dominated by that eye. (**B**) Ocular dominance is seen across the neuronal population. The left and middle graphs show histograms of the ocular dominance index at the end of the monocular and binocular phases of development, respectively. Most neurons have much the same response amplitudes at the end of the monocular phase whether stimulated through the left or right eye. Two contrasts are shown and differ in colour. The histogram has a much greater spread at the end of development, indicating that more cells are dominated by one eye or the other. This matches quite well with the five published histograms shown in the right graph; authors are represented by the first two letter of the first author’s name^2,37,38,39,40^. The match is better when the model’s stimulus contrast is 0.25, and this contrast is therefore used in the remainder of the figure. (**C**) The ocular dominance index at the end of development varies across the visual field. There are clusters of cells dominated by one eye or the other, consistent with the well-known columnar structure of ocular dominance. (**D**) This graph shows mean monocularity (1 for total domination by one eye, 0 for balanced input) at the end of development versus the interocular difference in preferred orientation at the end of monocular development. There is a significant correlation between the unaveraged quantities, suggesting that ocular dominance results from near-orthogonal preferred orientations at the end of the monocular development phase.

It has been known since the earliest studies of visual cortex that a substantial fraction of neurons in primary visual cortex are dominated by one eye or the other^36^. Figure 7A provides an example of ocular dominance in the model, using the neuron whose receptive field location is shown by the white circle in Fig. 7C. The graphs give orientation tuning curves at the end of monocular development (upper row) and the end of development (lower row). At the end of monocular development, the response amplitude when the cell is driven by the left eye is much the same as for right eye drive, but the preferred orientations are nearly orthogonal. At the end of binocular development, the preferred orientations are now nearly aligned but the response in the right eye is substantially less than that in the fellow eye.

Evidently, the development process cannot match the geniculocortical weights between eyes, and the left eye comes to dominate. The lack of amplitude growth for one eye is not due to cross-orientation suppression. Rather, it results from the same local process that shapes receptive fields in all cells: mutual cancellation between on-centre signals from one eye and off-centre signals from the other eye. The distinction for cells with orthogonal preferred orientations is that cancellation persists through much of the second development phase, and one eye suffers as a result.

Figure 7B shows that ocular dominance is a common property across the visual field. We calculated an ocular dominance index as described in the Methods; values of 0, 1 and 0.5 indicate a neuron dominated by the left, right and neither eye, respectively. The left and middle graphs show how ocular dominance changes during development. At the end of the monocular phase (left side) most cells have much the same drive from the two eyes. This is unsurprising given that the two eyes develop independently during this phase. As the end of development, however, there is a substantial fraction of neurons driven mainly by just one eye, as in the Fig. 7A. The histogram in this case falls mostly within the envelopes of five studies that measured ocular dominance, as shown at right. In general, these studies did not indicate stimulus contrast, which is why we used two contrasts in calculating the model’s response. The empirical data shows a bias towards dominance by the contralateral eye which is reflected in the model’s bias to the left eye. The bias in the model is, however, accidental: there is no monocular bias built into the model and model runs using differing seeds for the random number generator revealed no bias.

Figure 7C provides a map of the ocular dominance index across the visual field. There is a relatively smooth variation in ocular dominance with location, as found in previous work^41,42^. What is the origin of ocular dominance? A clue is provided in Fig. 7A: perhaps it is due to gross misalignment of orientation tuning at the end of monocular development. We test this hypothesis in Fig. 7D. The horizontal axis is the interocular difference in preferred orientation at the end of monocular development. The vertical axis is monocularity (defined in the Methods), where a value of 0 indicates an exact balance in the drive from the two eyes and a value of 1 indicates complete domination by one eye or the other. Monocularity is calculated at the end of development, binned, and averaged across all cells. There is a significant correlation between the unaveraged variables ($\rho =0.40,p=6.5\times {10}^{-38},n=961$), that is, a strong mismatch between preferred orientation at the end of monocular development often leads to ocular dominance.

## Discussion

### Summary and predictions

The timing of binocular congruence development in carnivores is well known. Neurons acquire orientation selectivity after the establishment of retinotopic projections^43^ and before or at the time of eye opening^6^. At the start of visual experience, however, the monocularly-driven orientation preference maps are uncorrelated^11^. Over the next week or so the maps become correlated. Also, at eye opening the eyes are strabismic^15^ and binocular disparity develops. The critical period for the maintenance of feature selectivity lasts about three weeks after eye opening^7^.

In this paper we have described a model that provides neuronal mechanisms underlying some of these events. The model proposes the following.

- Cortical responses are initially weak. They gain strength as spontaneous retinal waves of activity drive Hebbian plasticity in geniculocortical synapses. This plasticity up-regulates on- or off-centre inputs at each visual field location at the expense of the opposite sign of input. The resulting orientation preferences are uncorrelated between the eyes because stimulation is monocular.
- Eye opening sets off a new phase of Hebbian plasticity because stimulation is now binocular. In this case, responses at a specific visual field location are suboptimal because on-centre domination for stimulation through one eye can interfere with off-centre domination via the other eye. Plasticity improves response amplitude by up-regulating one eye’s inputs relative to the other. Orientation preference therefore becomes highly correlated between the eyes.
- Unstable binocular fixation due to strabismus exposes cortical neurons to variable binocular disparity. One disparity maximises the response in each cortical neuron because of differing on- and off-centre arrays in the two eyes, and Hebbian plasticity adjusts synaptic weights to match this preferred disparity. As a result, a range of preferred disparities develops across the neuronal population.
- When the eyes first open, the interocular difference between preferred orientations varies across the whole range from aligned to orthogonal. Plasticity cannot fully rescue differences close to orthogonal, but it improves neuronal responses by increasing synaptic weights from one eye relative to the fellow eye. The result is ocular dominance.

The model makes two key predictions. First, interocular on/off conflicts are resolved by synaptic plasticity in the period following eye opening. Second, where the interocular conflict is extreme—such as near-orthogonal orientation preferences—plasticity suppresses one eye’s input, resulting in ocular dominance. These predictions are not easy to test. Imaging techniques that can resolve neuronal activity at the level of single synaptic boutons are steadily evolving^44^, as is the stability of preparations capable of tracking day-to-day activity changes^11,13^. Testing the model’s predictions may be possible through these technological advances.

### Previous models

Our model is not the first to explore the development of binocular congruence. Berns et al.^45^ described a model in which Hebbian plasticity changed the weight of geniculocortical synapses. The model was simplified by assuming a visual field with a single spatial dimension. The particular interest of this model here is that development proceeded in two phases; activity in retinal ganglion cells was correlated in the same eye for the first phase, and between eyes for the second. Despite the constraints of the one-dimensional visual field, cortical neurons in the model displayed both a range of preferred binocular disparities and variable ocular dominance.

More recently, Chauhan et al.^46^ presented natural images to a binocular model that included geniculate cells, geniculocortical synapses with Hebbian plasticity, and excitatory cortical neurons. Cortical cells in the fully developed model exhibited binocular congruence of preferred orientation, and a spread of preferred binocular disparities. Binocular congruence can therefore develop in a model stimulated either with natural stimuli or, as in our own study, drifting gratings. Our model goes beyond the work of Chauhan et al. in that it provides mechanisms for the development of orientation selectivity before eye opening, and for the later development of binocular congruence with visual experience. We also show how ocular dominance can result from developmental processes prior to visual experience. Further, there is a good reason for using the same type of stimulus before and after eye opening: it allows quantitative comparison across the two stages of development (for example Fig. 4B).

### Orientation selectivity

Crair et al.^6^ used imaging of intrinsic optical signals in cat primary visual cortex to look for evidence of orientation selectivity several days after eye opening. They found spatial maps of preferred orientation like those in mature cortex, but the maps were more clearly defined for stimulation through the contralateral eye than through the ipsilateral. Our model replicates the finding of orientation maps early in visual experience (Fig. 5A) but does not reproduce the lateral bias. The earliest retinal projections are contralateral^47^, and this is presumably sufficient to establish the bias to contralateral stimulation. We have not implemented any such bias in the model because we wanted to keep the model as simple as possible. The Crair et al. study, incidentally, illustrates another finding that we will discuss in more detail below: there is no ocular dominance banding at the onset of visual experience.

Two studies^48,49^ used monocular eyelid suture to test whether binocular congruence can occur without shared visual experience. They found that monocularly driven maps of orientation preference were closely matched even though the eyes were never simultaneously open. These results appear, at first sight, to challenge the mechanism for binocular congruence that we have proposed in this paper. The period in which the first eye was open, however, was about a month long. It could therefore be that visual stimulation of a cortical neuron, in combination with spontaneous activity from the closed eye, could alter synaptic strengths sufficiently to binocularly align orientation preference. This issue clearly warrants further investigation.

### Binocular disparity

The development of binocular cooperation in the very young carnivore is difficult to study because of the weakness of binocular responses. Freeman and Ohzawa^50^ recorded from cortical cells in the cat two weeks after birth, that is, about one week after eye opening. They found binocular disparity tuning curves with similar shapes to those in the adult, but with substantially lower impulse rates. Thereafter, response amplitude improved steadily with age. Responses in the model (Fig. 4C) are consistent with these findings.

### Ocular dominance

We have shown that the model, when fully developed, includes a substantial fraction of cortical neurons that are driven more by one eye than by the other. We have also shown that histograms of ocular dominance are like those in the literature. How does this ocular dominance arise? Chang et al.^11^, in their study of visual development in the ferret, tracked neurons for four days after eye opening and found that, first, the interocular mismatch between preferred orientations diminishes dramatically and, second, there is a modest increase in ocular dominance in that period. They did not, however, find a significant correlation between initial orientation mismatch and later ocular dominance.

Our results are consistent with those of Chang et al. in that we find both binocular alignment of preferred orientation (Fig. 5B) and an increase in ocular dominance (Fig. 7) during the binocular stimulation phase of development. We differ in finding a significant correlation between the orientation mismatch at the start of this phase with the ocular dominance at the end (Fig. 7D). The reason for this disagreement between results could be timing. Chang et al. tracked their neurons for four days whereas the plastic period for ferret visual cortex extends to three weeks. Our hypothesis, then, is that ocular dominance is a sequel to large interocular differences in preferred orientation before visual experience. Horton and Adams^41^ argued that ocular dominance columns serve no visual purpose. We have described how ocular dominance could originate as a by-product of processes that contribute to visual perception.

The time at which ocular dominance columns develop in the carnivore is still somewhat controversial. Crair et al.^51^ injected a tracer into one eye of the cat and found a periodic label at day P14 (about a week after eye opening), but not at P7. It has been shown, however, that transneuronal tracers tend to spread between the monocular layers of the lateral geniculate in very young animals^52^. Crair et al. added controls to circumvent this problem, such as flattening the cortex to obtain maximum sensitivity for patterned tracer concentration. Further, Chang et al.^11^, recording from ferret cortex immediately after eye opening, found very little evidence of ocular dominance (mean monocularity = 0.14). On the balance of evidence, therefore, it seems that ocular dominance columns develop soon after eye opening. Our model is consistent with these previous results.

### Intracortical connections

Intracortical synaptic connections are ubiquitous in primary visual cortex^53^. The intracortical connections implemented in our model are all inhibitory: half of the cortical neurons are inhibitory, and each inhibitory neuron connects to all excitatory neurons. We have not explicitly described inhibitory influences in the results, but they play a crucial role in refining orientation selectivity via the iceberg effect^16^. See, for example, the orientation tuning curves in Fig. 4B. The mean orientation bandwidth (half-width at half-height) is 20° and 21° at the end of the monocular and binocular development phases, respectively. Excitatory-to-excitatory connections are also prevalent in cat primary visual cortex^53^. We have excluded these connections here to keep the focus on binocular mechanisms, but have shown elsewhere^17^ that adding excitatory intracortical connections enhances essential model properties.

## Supplementary Information

Supplementary Information.

## Data Availability

The software used to run the model is provided in the Supplementary information.
