# Supplementary material for: A model for the development of binocular congruence in primary visual cortex
Source: Sci Rep. 2022 Jul 25;12:12669. doi: 10.1038/s41598-022-16739-6 (PMC9314406; doi:10.1038/s41598-022-16739-6)
Supplement: Supplementary file 1 — Supplementary Information. [file 41598_2022_16739_MOESM1_ESM.zip › Code/Guide to anaTab.pdf]

Guide to anaTab

anaTab is a Matlab function that enables the sequential analysis of tabular data. There are two types of sequence.

1. There is a sequence of analysis tasks, such as 'select plot set'. This sequence selects a subset of the data, plots it and formats the plot.
2. For each task, the data table is split into groups which are analysed in turn. A group is defined by a set of variables in the data table, such as {'subject', 'contrast'}. In this example a group is a set of rows in the table representing a single subject and contrast.

Data are contained in two variables d and m, which stand for data table and metadata, respectively. A column in d represents a variable such as subject, and a row in d is a single observation. The metadata variable m indicates how the analysis is to be performed. For example, m.select.subject = 'AB' indicates that the select task is to keep only those data table rows for which the subject is AB. Task *task* is typically performed with the function call [d, m] = do*Task*(d, m), which transforms the input data table on the right into the table on the left under the instructions of the metadata on the right.

anaTab uses a number of variables:

- d is the data table
- m is the metadata
- m.group is the number of the group currently being analysed
- m.groups is the number of groups for the current task
- m.tasks is the sequence of tasks to be performed
- m.*task*.fun is the function used to execute *task*

- m.*task*.group is a list of the table variables that are constant within each group analysed by *task*
- m.*task*.arg is a value used by *task*
- m.x and m.y are the independent and dependent variables, respectively, in the analysis
- m.handle.axes, m.model are internal variables

Limited help on anaTab can be obtained with help anaTab. Some task functions, such as doSelect, include a description of their inputs and outputs.
