# Supplementary material for: A model for the development of binocular congruence in primary visual cortex
Source: Sci Rep. 2022 Jul 25;12:12669. doi: 10.1038/s41598-022-16739-6 (PMC9314406; doi:10.1038/s41598-022-16739-6)
Supplement: Supplementary file 1 — Supplementary Information. [file 41598_2022_16739_MOESM1_ESM.zip › Code/Read me.rtf]

This folder contains the code for a model of signal processing in the upstream visual system.You will also need the file of geniculocortical synaptic weights: https://drive.google.com/file/d/1gXFzkxhlyYERT-NTB1AMjMqcNVKbOtzh/view?usp=sharing.The code runs in Matlab. Ensure all .m and .mat files are on the Matlab path.Run anaBin. This should plot a map of synaptic weights.Now open anaBin. The first switch statement controls the analysis. You just ran case weight.x.y: try some other cases.All cases set metadata used by anaTab, which executes the analyses. See Guide to anaTab and anaTabTute.m for short tutorials on anaTab.
